# Retraction Note: Enhancement of cardiac angiogenesis in a myocardial infarction rat model using selenium alone and in combination with PTXF: the role of Akt/HIF-1α signaling pathway

**DOI:** 10.1007/s00210-025-03935-0

**Published:** 2025-03-03

**Authors:** Mohamed M. Elseweidy, Sousou I. Ali, Mohamed A. Shaheen, Asmaa M. Abdelghafour, Sally K. Hammad

**Affiliations:** 1 Biochemistry Department, Faculty of Pharmacy, Zagazig University, Zagazig, 44519 Egypt; 2 Histology and Cell Biology Department, Faculty of Human Medicine, Zagazig University, Zagazig, 44519 Egypt

**Retraction Note: Naunyn-Schmiedeberg's Archives of Pharmacology (2023) 397:4677–4692**

10.1007/s00210-023-02904-9

The Editor-in-Chief has retracted this article. A number of concerns were raised regarding the similarity in the datasets of this manuscript, and in [1]. The authors provided their raw data, but their explanation that their similarity could be attributed to using small cohorts did not assuage concerns. Furthermore:

- The trace for Fig. 2A Se + PTXF was found to overlap with the trace for Fig. 2A Van + PTXF
- Figure 8D was found to overlap with 6E in [1]
- A number of duplicated artifacts were found within the same panels of Fig. 7C, D, and E, and within Fig. 8A.

The Editor-in-Chief has lost confidence in the data and conclusions of this article.

All authors disagree with this retraction.
